# Circ‐SERPINE2 promotes the development of gastric carcinoma by sponging miR‐375 and modulating *YWHAZ*

**DOI:** 10.1111/cpr.12648

**Published:** 2019-06-14

**Authors:** Jianing Liu, Suzhen Song, Sen Lin, Mingbao Zhang, Yating Du, Dongdong Zhang, Weihua Xu, Hongbo Wang

**Affiliations:** ^1^ Department of Thyroid and Pancreatic Disease The Second Hospital of Shandong University Jinan China; ^2^ Department of Internal Medicine Shandong University of Traditional Chinese Medicine Jinan China; ^3^ Department of Digestive Disease The Second Hospital of Shandong University Jinan China

**Keywords:** 14‐3‐3 zeta, circular RNA, gastric carcinoma, miR‐375, *YWHAZ*

## Abstract

**Objectives:**

Circular RNAs (circRNAs) exist extensively in the eukaryotic genome. The study aimed to identify the role of hsa_circ_0008365 (Circ‐SERPINE2) in gastric carcinoma (GC) cells and its downstream mechanisms.

**Materials and methods:**

Gene Expression Omnibus (GEO) database was applied to screen differentially expressed circRNAs. CircInteractome, TargetScan and miRecords websites were used to predict target relationships. qRT‐PCR and RNase R treatment were utilised to detect molecule expression and confirm the existence of circ‐SERPINE2. RNA pull‐down assay and dual‐luciferase reporter assay were performed for interaction between circRNA and miRNA or mRNA. EdU assay, colony formation assay, and flow cytometry for apoptosis and cell cycle detections were utilised to assess cell function. Western blot and immunohistochemistry (IHC) assays were applied for detection of proteins in tissues or cells.

**Results:**

Circ‐SERPINE2 and *YWHAZ* were upregulated, and miR‐375 was downregulated in GC tissues and cells. Circ‐SERPINE2 and *YWHAZ* targetedly bound to miR‐375. Circ‐SERPINE2 promoted cell proliferation and cell cycle progress and inhibited cell apoptosis by sponging miR‐375 and regulating *YWHAZ* expression in vitro. Circ‐SERPINE2 repressed solid tumour growth through enhancing miR‐375 expression and reducing *YWHAZ* expression in vivo.

**Conclusions:**

Circ‐SERPINE2 is a novel proliferative promoter through the regulation of miR‐375/*YWHAZ*. Circ‐SERPINE2/miR‐375/*YWHAZ* axis might provide a novel therapeutic target of GC.

## 1 INTRODUCTION

Gastric cancer (GC) is one of the most widespread malignancies. In China, the mortality rate of GC is ranked the first place among several malignancies.^1^ The lymph node metastasis and the haematogenous metastasis are the main two routes for GC translocation.^2^ At present, it is recognised that the gastroscopy is conducive to the early detection and diagnosis of GC. Surgery is still the only treatment strategy for GC.^3^ Due to the high recurrence rate of GC, the 5 year overall survival rate is less than 30%.^4^ Hence, it is urgent to further survey the molecular mechanism of GC and find the potential biomarker.

Researches related to circular RNAs (circRNAs) have been around for nearly forty years. However, until recent years, scholars began to pay more attention to them.^5^ Their highly stable expression and distribution in various tissues suggest that circRNAs have a vital impact on human disease.^6^ Studies have shown that circRNAs have a powerful regulatory function in cancer,^4^ especially in GC. The study of Zhao et al^7^ showed that the hsa_circ_0000181 expression level was much higher in GC tissues than that in normal control tissues. Its expression in GC was related to tumour diameter, lymphatic metastasis and distal metastasis. Furthermore, Xie et al^8^ found that the hsa_circ_0074362 expression was observably increased in cells and tissues of GC, and the gastric cancer lymphatic metastasis was positively correlated with the hsa_circ_0074362 expression level.

microRNAs (miRNAs) are a kind of non‐coding RNA with a length of 18‐25 nucleotides, and the abnormal expression of miRNA is closely related to various diseases, especially cancer.^9^ Several researches have shown that miRNAs are involved in cell proliferation and apoptosis in many malignant tumours, such as colon cancers,^10^ melanoma^11^ and renal cancer.^12^ Chang et al^13^ found that the expression of miR‐375 had significant difference in the GC tissues with or without *Helicobacter pylori* infection. Xu et al^14^ revealed that miR‐375 was regulated by *Snail* and targeted *JAK2*, ultimately inhibiting the invasion and migration of GC cells. However, the interaction mechanisms of circRNAs and miRNA are waiting for be further explored in GC.

*YWHAZ*, also known as 14‐3‐3 zeta, belongs to one member of 14‐3‐3 protein family, which is highly conservative in all eukaryotes and can regulate many cell events, such as signal transduction, cell cycle and apoptosis.^15^ Recently, *YWHAZ* has been identified as a clinical prognostic indicator for some tumours.^16^ The study of Bergamaschi et al^17^ showed that 14‐3‐3 zeta was a key predictor for risk of failure in endocrine therapy and was valuable in recovering endocrine sensitivity and decreasing recurrence risk in breast cancer. Nishimura et al illustrated that the YWHAZ protein expression was linked to the tumour size, lymphatic and venous invasion, tumour depth, pathological stage and recurrence rate. Besides, the higher level of YWHAZ protein was significantly correlated with the lower level of miR‐375 expression.^16^ However, the research on the upstream molecular mechanism of *YWHAZ* in GC was limited, which prompts us to conduct a more in‐depth study on the molecular mechanism of *YWHAZ* in GC.

In tumour development, circRNA‐miRNA‐mRNA interaction networks may be involved and play a crucial role. Here, we aimed to explore how hsa_circ_0008365 (circ‐SERPINE2) exerts its function in GC through exploring the correlation among circ‐SERPINE2, miR‐375 and *YWHAZ* and their expressions in GC tissues and cells. Furthermore, the findings from in vitro experiments were verified by in vivo experiments. This study might provide a new molecular marker for the molecular therapy of GC.

## 2 MATERIALS AND METHODS

### 2.1 Patient samples

Tissues and adjacent tissues of GC samples were obtained from 49 GC patients in The Second Hospital of Shandong University from February 2012 to February 2017. National Comprehensive Cancer Network (NCCN) Oncology Clinical Practice Guidance (V.1.2012) and tumour–nodes–metastasis (TNM) staging system were utilised to make a definite diagnosis. Meanwhile, GC was identified by histopathology. Human Research Ethics Committee approved this study. Besides, this study was supported with written informed consent obtained from all enrolled subjects.

### 2.2 Bioinformatics analysis

GSE78092 (based on GPL21485 platform) containing three cases of GC tissues and three cases of its adjacent tissues and GSE93541 (based on GPL19978 platform) containing six total RNA data extracted from three plasma samples of gastric cancer patients and three healthy controls are obtained from Gene Expression Omnibus (GEO) database to analyse differentially expressed circRNAs in R 3.4.1 software. The present study presented the top 100 differentially expressed circRNAs using pheatmaps with log|fold change| > 1 and adjusted *P* value < 0.05 by microarray analysis. Subsequently, circRNA and miRNA interactions were predicted using CircInteractome website (https://circinteractome.nia.nih.gov/); and miRNA and mRNA target relationship was supported by TargetScanHuman 7.2 (http://www.targetscan.org/vert_72/). Furthermore, miRNAs revealed in HMDD v3.0 (http://www.cuilab.cn/hmdd) were consulted. The genes targeted hsa‐miR‐375 were sought in miRecords website (http://c1.accurascience.com/miRecords/index.php). In addition, Venny 2.1 (http://bioinfogp.cnb.csic.es/tools/venny/) was utilised to reveal the intersection among various types of subset.

### 2.3 Cell culture

Human gastric smooth muscle cells (HGSMC) and human GC cells (AZ521 and MGC‐803) were cultured in 90% Dulbecco's Modified Eagle's Medium‐High Glucose (DMEM‐H) supplemented with 10% foetal bovine serum (FBS). Human gastric mucosa cells (GES 1), human GC cells (HGC‐27) and human embryonic kidney cells (HEK 293T) were cultured in 90% RPMI‐1640 and 90% RPMI‐1640 (w/o HEPES) supplemented with 10% FBS, respectively. Additionally, all cell lines were purchased from BeNa Culture Collection (BNCC) and maintained in the atmosphere of 5% CO_2_ and 37°C.

### 2.4 RNase R treatment and qRT‐PCR

Total RNA from tissues or cell lines was isolated by TRIzol reagent (Life Technologies) with RNeasy Mini Kit (QIAGEN). After RNase R treatment, genomic DNA (gDNA) was isolated using QIAamp DNA Mini Kit (QIAGEN). Quantitative real‐time PCR (qRT‐PCR) was conducted with the SYBR Premix Ex TaqTM kit (TaKaRa). The primer sequences used for qRT‐PCR were displayed in Table 1. The expressions of miRNA and circRNA/mRNA were normalised to the levels of U6 and GAPDH and calculated with the 2^−ΔΔCt^ method.

**Table 1:** Primer sequences for qRT‐PCR

Genes	Sequences	
circ‐SERPINE2	Forward primer	5′‐CCCGAGAACACAAAGAAACGC‐3′
	Reverse primer	5′‐AAGAGGGGGAGATGCCAGTT‐3′
linear SERPINE2	Forward primer	5′‐CATCCCACACATCAGCACCA‐3′
	Reverse primer	5′‐AGGTTTTCTGACCCTGTTGTTA‐3′
YWHAZ	Forward primer	5′‐TTTCTCCTTCCCCTTCTTCCG‐3′
	Reverse primer	5′‐GCCAGTTTGGCCTTCTGAAC‐3′
GAPDH	Forward primer	5′‐TCGGAGTCAACGGATTTGGT‐3′
	Reverse primer	5′‐TTCCCGTTCTCAGCCTTGAC‐3′
Divergent GAPDH	Forward primer	5′‐GAAGGTGAAGGTCGAGTC‐3′
	Reverse primer	5′‐GAAGATGGTGATGGGATTTC‐3′
miR‐375	Forward primer	5′‐AAGCTTTGTTCGTTCGGCTC‐3′
	Reverse primer	5′‐GTATCCAGTGCGAATACCTC‐3′
miR‐203	Forward primer	5′‐TGCTCTAGAGGCGTCTAAGGCGTCCG‐3′
	Reverse primer	5′‐CCCAAGCTTCACCTCCCAGCAGCACTTG‐3′
miR‐194	Forward primer	5′‐ATGGACCTGGGGCCAGCGAAG‐3′
	Reverse primer	5′‐TCTGGCCTGGGAGCGTCG‐3′
U6	Forward primer	5′‐CTCGCTTCGGCAGCACA‐3′
	Reverse primer	5′‐AACGCTTCACGAATTTGCGT‐3′

### 2.5 Plasmid construction and stable transfection

Human circ‐SERPINE2 cDNA was synthesised and provided by TSINGKE and then cloned into pcD‐ciR vector (Geneseed Biotech) to construct circ‐SERPINE2 overexpression plasmids. Then, transfection was implemented with these plasmids and GC cells. Then, the transfected cells were selected with G418 (Life Technologies).

### 2.6 Oligonucleotide transfection

Short interfering RNAs (siRNAs) for hsa_circ_0008365 (circ‐SERPINE2) knockdown were designed by CircInteractome web and synthesised by RiboBio. The siRNA against for *YWHAZ* (si‐YWHAZ), miR‐375 mimics, miR‐375 inhibitor, universal siRNA negative control (si‐NC), mimics control (mimics‐NC) and inhibitor control (inhibitor‐NC) were provided by RiboBio. The sequences of siRNAs were displayed in Table 2.

**Table 2:** The sequences of siRNAs

siRNAs	Sequences
si‐circ‐SERPINE2‐1	5′‐GTGTGGTCGTCCTTGGTGGAA‐3′
si‐circ‐SERPINE2‐2	5′‐TGTTCCGGTGTGGTCGTCCTT‐3′
si‐YWHAZ	5′‐GATGACATGGCAGCCTGCATGAAGT‐3′
si‐NC	5′‐UUCUCCGAACGUGUCACGUTT‐3′

Abbreviation: NC, negative control.

### 2.7 RNA immunoprecipitation

RNA immunoprecipitation (RIP) assays were performed using a Magna RIP Kit (Millipore) following the manufacturer′s instructions. In brief, miR‐375 mimics and control mimics were transfected into HGC‐27 and MGC‐803 cells. 2 × 10^7^ HGC‐27 and MGC‐803 cells were lysed in 100 μL RIP lysis buffer supplemented with RNase inhibitor (Promega) and protease inhibitor cocktail (Roche). After treated with DNase I (Roche), the lysate was then diluted with 900 μL RIP immunoprecipitation buffer and incubated with antibody‐coupled magnetic beads (anti‐AGO2, Abcam). RNA immunoprecipitation mixture (10 μL) was saved as input. Beads were subsequently washed six times with RIP wash buffer and treated with proteinase K at 37°C for 30 minutes. RNA was extracted using TRIzol reagent (Invitrogen) according to the manufacturer′s instructions.

### 2.8 Pull‐down assay with biotinylated‐circ‐SERPINE2 probe

A total of 1 × 10^7^ GC cells were collected, lysed and sonicated. Biotinylated‐circ‐SERPINE2 probe was synthesised and provided by RiboBio. The circ‐SERPINE2 probe was incubated at 25°C for 2 hour with C‐1 magnetic beads (Life Technologies), and the cell lysates were incubated at 4°C overnight with oligo probe or circ‐SERPINE2 probe. The RNA complexes combined with the beads were extracted using RNeasy Mini Kit (QIAGEN) for RT‐PCR.

### 2.9 Pull‐down assay with biotinylated miR‐375

Biotinylated miR‐375 mimics and its mutant were synthesised by RiboBio. The stably expressed circ‐SERPINE2 GC cells were transfected with 50 nM of biotinylated miR‐375 mimics or mutant using Lipofectamine RNAiMax (Life Technologies), harvested after transfection for 48 hour and followed by sonication. The cell lysates (50 μL) were aliquot for input, and the remanent cell lysates were incubated at 4°C for 3 hour with C‐1 magnetic beads (Life Technologies) and then washed by wash buffer. The combined RNAs were purified with RNeasy Mini Kit (QIAGEN) for RT‐PCR.

### 2.10 Luciferase reporter assay

The circ‐SERPINE2 or *YWHAZ* 3′UTR containing miR‐375 binding sites were synthesised and subcloned into a pGL2‐Base vector (Promega). HEK‐293T cells were plated into 96‐well plates and were co‐transfected with a mixture of firefly luciferase reporter, pRL‐CMV Renilla luciferase reporter, and miR‐375 mimics or its mutant control using Lipofectamine 2000 (Invitrogen). After 48 hour of incubation, the firefly and Renilla luciferase activities were quantified with a dual‐luciferase reporter assay system (Promega). The Renilla luciferase activity was considered as internal control.

### 2.11 5‐Ethynyl‐2′‐deoxyuridine (EdU) assay

An EdU assay kit (RiboBio) was used to detect DNA synthesis and cell proliferation. Transfected GC cells (1 × 10^4^) were seeded into 96‐well plates for one night. The next day, cells were incubated with Edu solution (50 μM) for 2 hour at 37°C. Afterwards, cells were fixed with 4% paraformaldehyde for 30 minutes and incubated with glycine (2 mg/mL) for 5 minutes. In the following step, cells were permeabilised with PBS of 0.5% Triton X‐100 for 10 minutes and then stained using Apollo reaction solution for 30 minutes. Cells were stained with Hoechst for 30 minutes in the dark after washed several times with PBS of 0.5% Triton X‐100. Finally, cells were imaged by a Nikon microscope (Nikon) and the percentage of EdU‐positive cells from four random fields in three wells was calculated.

### 2.12 Western blot

The concentration of protein was measured by the bicinchoninic acid (BCA) method. Samples with equal amounts of protein were divided via SDS‐PAGE and transferred onto the membranes of PVDF (Millipore). After blocking with 5% skim milk, incubation with primary antibody anti‐Bcl‐2 (1:1000, Abcam), anti‐Bax (1:5000, Abcam), anti‐PCNA (1:5000, Abcam), anti‐14‐3‐3 zeta (namely anti‐YWAHZ, 1:1000, Abcam) and internal control anti‐GAPDH (1:5000, Abcam) at 4°C overnight were performed. The membranes were incubated with secondary antibody (HRP‐conjugated) at room temperature for 1 hour. Immunoreactive bands were visualised using an enhanced chemiluminescence kit (Millipore).

### 2.13 Colony formation assay

Within the colony formation assays, the transfected cells were trypsinised, plated in 24‐well plates and incubated routinely at 37°C for 14 days. Colonies were fixed using methanol for 10 minutes and dyed with 0.1% crystal violet for 15 minutes. Cell colonies were then counted and analysed.

### 2.14 Flow cytometry assays

Annexin‐V‐FITC/Propidium Iodide (PI) Apoptosis Detection Kit (BD Biosciences) was applied. The transfected GC cells were stained using FITC and PI (BD Pharmingen). The fluorescence‐activated cell sorting was conducted by FACScan (BD Biosciences). The analysis of cell apoptosis was performed using Flowjo V10 software (Tree Star). The transfected GC cells were stained with PI buffer (BD Pharmingen). Cell cycle data were analysed using the ModFit LT software.

### 2.15 Immunohistochemistry (IHC)

The paraffin sections (5 μM) of tumour tissue samples or adjacent tissue samples were dehydrated using the graded ethanol and incubated. After blocking endogenous peroxidase, the sections were incubated with primary antibody anti‐14‐3‐3 zeta (ab51129, 1:1000, rabbit anti‐human, Abcam) or rabbit IgG isotype at 4°C overnight. Then, the sections were washed with TBS wash buffer and incubated with secondary antibody (goat anti‐rabbit). The 14‐3‐3 zeta immunoreactivity was scored by three pathologists based on staining intensity and staining distribution. Scores 0, 1, 2 and 3 were for negative staining, faint yellow staining, sandy beige staining and dark brown staining, respectively. Meanwhile, scores 1, 2, 3 and 4 were for 0%‐25% staining, 25%‐50% staining, 50%‐75% staining and 75%‐100% staining, respectively. The final score was the sum of staining intensity and distribution score.

### 2.16 Xenograft model

All protocols of animal experiments were approved by the Animal Care Committee of The Second Hospital of Shandong University. Four‐week‐old female BALB/C nude mice purchased from Guangdong Medical Laboratory Animal Center were randomly divided into 2 groups (n = 5 for each group). The GC cells stably transfected the mixture of si‐circ‐1 and AreloGene® (Boppard, used to stably downregulate circ‐SERPINE2 in vivo) or control vector (5 × 10^6^ cells per nude mouse) were subcutaneously injected into the right flank of nude mice. Tumour sizes were calculated with the formula 1/2 (length × width^2^). After monitoring for 4 weeks, mice were sacrificed and tumours were separated for further analyses.

### 2.17 Statistical analysis

GraphPad Prism (Version 6.0) was utilised to perform the statistical analyses, in which data were documented as means ± standard deviation. Student′s *t* test or one‐way ANOVA was applied to examine the statistical significance for the comparison of two or more groups with *P* < 0.05.

## 3 RESULTS

### 3.1 Circ‐SERPINE2 is upregulated in the microarray analysis of human GC

**Figure 1:**
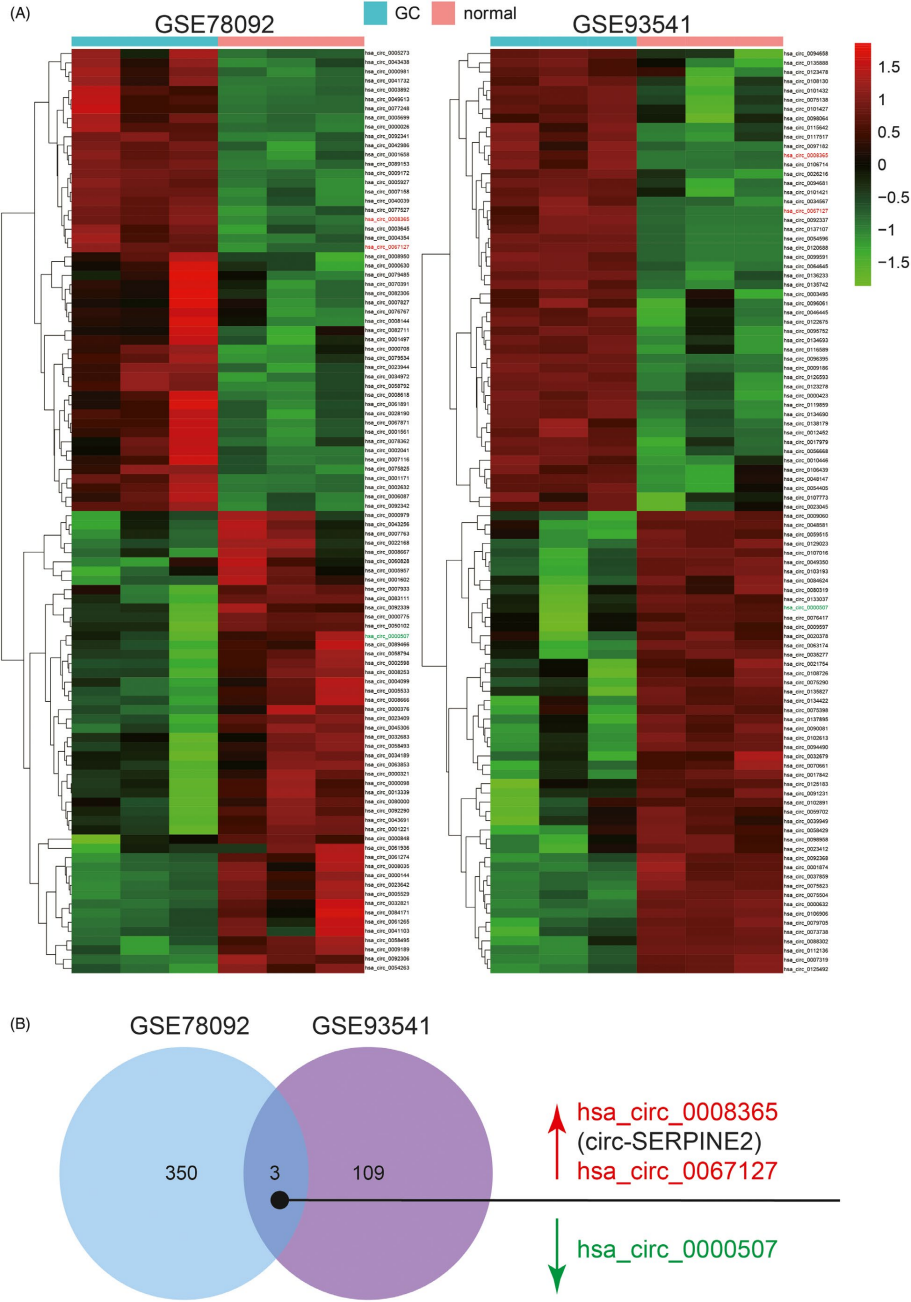
Differential expression profiles of circRNAs in the human gastric cancer and normal control samples. A, Top 100 differentially expressed circRNAs between the GC and the normal control samples in GSE78092 and in GSE93541 based on the criteria of log|fold change|>1 and adjusted *P* value < 0.05. CircRNAs written in red indicated the expression upregulation, and green indicated the expression downregulation. B, The common differentially expressed circRNAs in GSE78092 and in GSE93541 were revealed using Venn diagram. Hsa_circ_0008365/circ‐SERPINE2 was upregulated in GC. CircRNAs written in red indicated the expression upregulation, and green indicated the expression downregulation

According to the GEO database of GC, two microarray chips, namely GSE78092 and GSE93541, were selected to filtered differentially expressed circRNAs. With the criteria of log|fold change| > 1 and adjusted *P* value < 0.05, top 100 differentially expressed circRNAs between the GC and the normal control samples in GSE78092 and in GSE93541 were displayed by heat map in Figure 1A, respectively. The common differentially expressed circRNAs in GSE78092 and in GSE93541 were revealed using Venn diagram. Two circRNAs with expression upregulation in GC (namely hsa_circ_0008365/circ‐SERPINE2 and hsa_circ_0067127/circ‐ALDH1L1) and the hsa_circ_0000507 (circ‐CUL4A) with expression downregulation in GC were found (Figure 1B). The detailed log|fold change| and adjusted *P* value of three circRNAs in GSE78092 and GSE93541 are shown in Table S1. Among the 3 screened circRNAs, only circ‐SERPINE2 is predicted to bind to AGO2 (Table S2), a protein plays a central role in RNA silencing processes, as an essential catalytic component of the RNA‐induced silencing complex (RISC).

### 3.2 Circ‐SERPINE2 is upregulated and can sponge miR‐375 in GC tissues and cells

**Figure 2:**
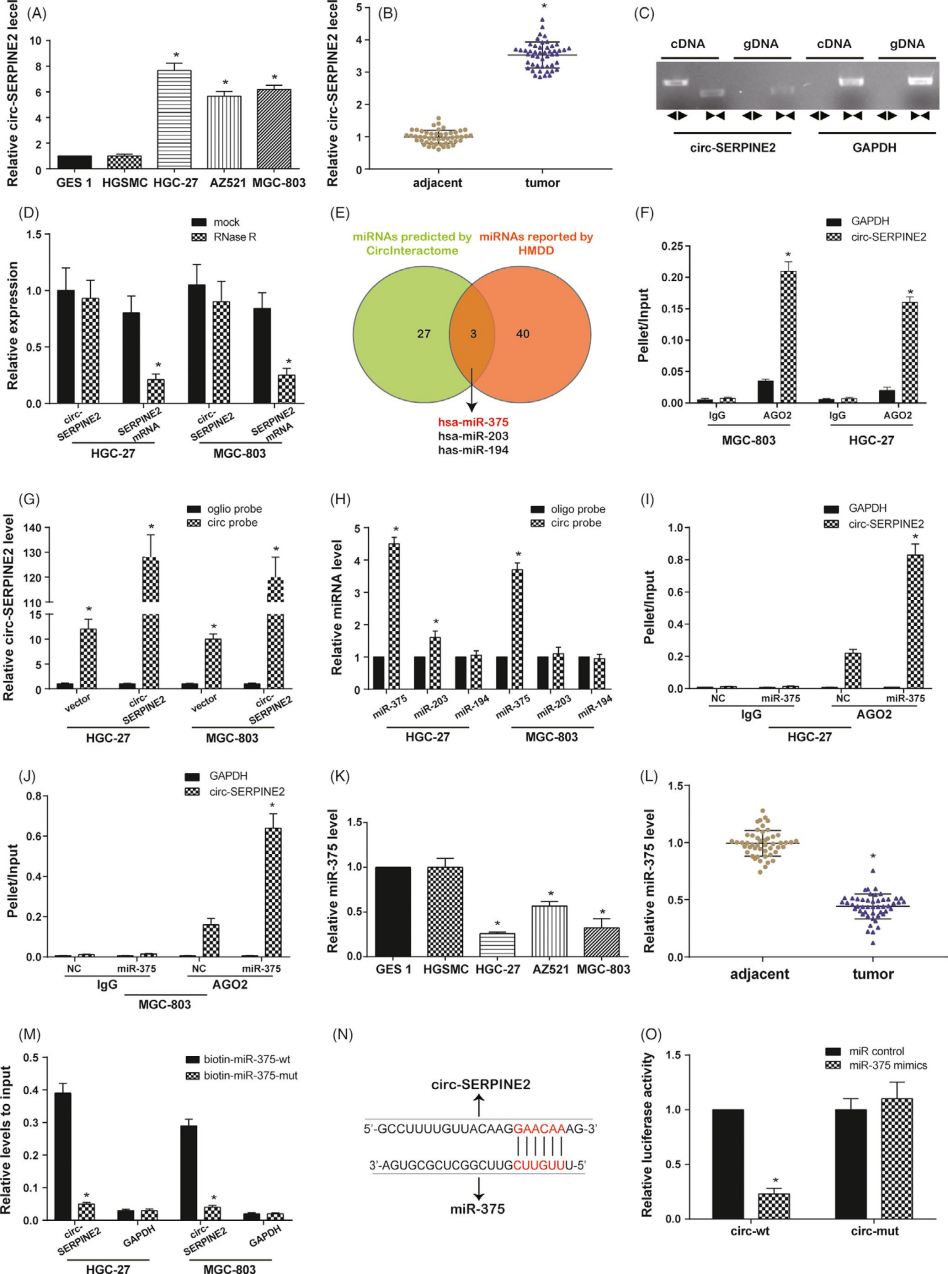
Circ‐SERPINE2 expression and validation in GC tissues and cells; circ‐SERPINE2 sponges miR‐375 in GC tissues and cells. A, B, qRT‐PCR assay suggested the expression of circ‐SERPINE2 was upregulated in GC cell lines and tissues (n = 49). C, RT‐PCR assay with divergent or convergent primers indicated the existence of circ‐SERPINE2 in GC tissue. GAPDH was used as negative control. D, qRT‐PCR analysis of the expression of circ‐SERPINE2 after RNase R treatment. E, The intersection of miRNAs targeted circ‐SERPINE2 predicted by CircInteractome and miRNAs related to GC reported in HMDD is shown via Venn diagram. F, Immunoprecipitation of AGO (control, mouse IgG) in HGC‐27 and MGC‐803 cells was performed. G, Lysates from HGC‐27 and MGC‐803 cells with circ‐SERPINE2 overexpression were subjected to biotinylated‐circSERPINE2 pull‐down assay, and the expression levels of circ‐SERPINE2 were measured by qRT‐PCR. H, The expression levels of three candidate miRNAs shown in E were quantified by qRT‐PCR after the biotinylated‐circ‐SERPINE2 pull‐down assay in HGC‐27 and MGC‐803 cells. I, J, Immunoprecipitation of AGO2 (control, mouse IgG) in miR‐375 mimics or control mimics (NC)‐transfected HGC‐27 and MGC‐803 cells were performed. K, L, qRT‐PCR assay suggested the expression of miR‐375 was downregulated in GC cell lines and tissues (n = 49). M, The biotinylated wild‐type/mutant miR‐375 was, respectively, transfected into HGC‐27 and MGC‐803 cells with circ‐SERPINE2 overexpression. The expression levels of circ‐SERPINE2 were tested by qRT‐PCR after streptavidin capture. N, The binding site between circ‐SERPINE2 and miR‐375 predicted in CircInteractome. O, Luciferase activity in HEK 293T cells co‐transfected with luciferase reporter containing circ‐SERPINE2 sequences with wild‐type or mutated miR‐375 binding sites and the mimics of miR‐375 or control. Data were represented as means ± SD of at least three independent experiments. *P < 0.05

GC cell lines were used to perform the further experiments, including GES 1, HGSMC and gastric cancer cell lines (HGC‐27, AZ521 and MGC‐803). The qRT‐PCR analysis showed that circ‐SERPINE2 has higher expression in GC cells than in normal control cells (Figure 2A). Clinical GC patients′ tissues and adjacent tissues confirmed circ‐SERPINE2 was upregulated in GC (Figure 2B). To verify the head‐to‐tail splicing, on the one hand, the divergent primers and convergent primers were designed to amplify circular RNA and linear RNA based on cDNA and gDNA from GC tissues. As shown in Figure 2C, circ‐SERPINE2 was amplified in cDNA by two primers, but not in gDNA with the divergent primer. On the other hand, RNase R treatment was conducted. After RNase R treatment, the RNAs of circular form resisted to RNase R, but the linear RNAs was significantly reduced (Figure 2D).

CircInteractome website was used to predict target miRNAs of circ‐SERPINE2, and miRNAs related to GC reported in HMDD v3.0 were revealed. As shown in Figure 2E, the overlapping of foregoing two types of miRNAs contained hsa‐miR‐375, hsa‐miR‐194 and hsa‐miR‐203. To assess whether circ‐SERPINE2 acts as a sponge for miRNAs, we performed AGO2 immunoprecipitation using the lysates of HGC‐27 and MGC‐803 cells. RNA immunoprecipitation (RIP) assays showed that circ‐SERPINE2 was enriched in AGO2 immunoprecipitation, suggesting that circ‐SERPINE2 might have interactions with miRNAs (Figure 2F). Then, a 3′‐terminal‐biotinylated‐circ‐SERPINE2 probe was designed to address which miRNAs latently interact with circ‐SERPINE2. The probe was confirmed to pull‐down circ‐SERPINE2 in GC cells. Meanwhile, circ‐SERPINE2 overexpression enhanced the pull‐down efficiency (Figure 2G). After pull‐down assay, three candidate miRNAs were measured by qRT‐PCR, and miR‐375 was the only one miRNA abundantly pulled down by circ‐SERPINE2 probe in both HGC‐27 and MGC‐803 cells (Figure 2H). AGO2 immunoprecipitation also demonstrated the association of miR‐375 with circ‐SERPINE2 (Figure 2I,J). Taken together, these results suggest that circ‐SERPINE2 may exert its function through direct interaction with miR‐375.

Therefore, the potential interactions between circ‐SERPINE2 and miR‐375 were further explored. Firstly, miR‐375 expressions were assessed in GC cells and tissues. The results suggested that, opposite to circ‐SERPINE2, miR‐375 was downregulated in GC cells and tissues (Figure 2K,L). Next, HGC‐27 and MGC‐803 cells with stable circ‐SERPINE2 overexpression were transfected with biotinylated miR‐375 or its mutant. The binding of circ‐SERPINE2 and the miR‐375 or its mutant was detected by qRT‐PCR. A higher enrichment in the group of wild‐type miR‐375 (biotin‐miR‐375‐wt) was observed compared with the group of mutant miR‐375 (biotin‐miR‐375‐mut) (Figure 2M). Furthermore, binding site between circ‐SERPINE2 and miR‐375 predicted in CircInteractome is shown in Figure 2N. In addition, dual‐luciferase reporter assay further verified circ‐SERPINE2 could directly bind to miR‐375 (Figure 2O). Above all, these findings indicated circ‐SERPINE2 was upregulated and could sponge miR‐375 in GC tissues and cells.

### 3.3 Circ‐SERPINE2 promotes GC cells development by sponging miR‐375

**Figure 3:**
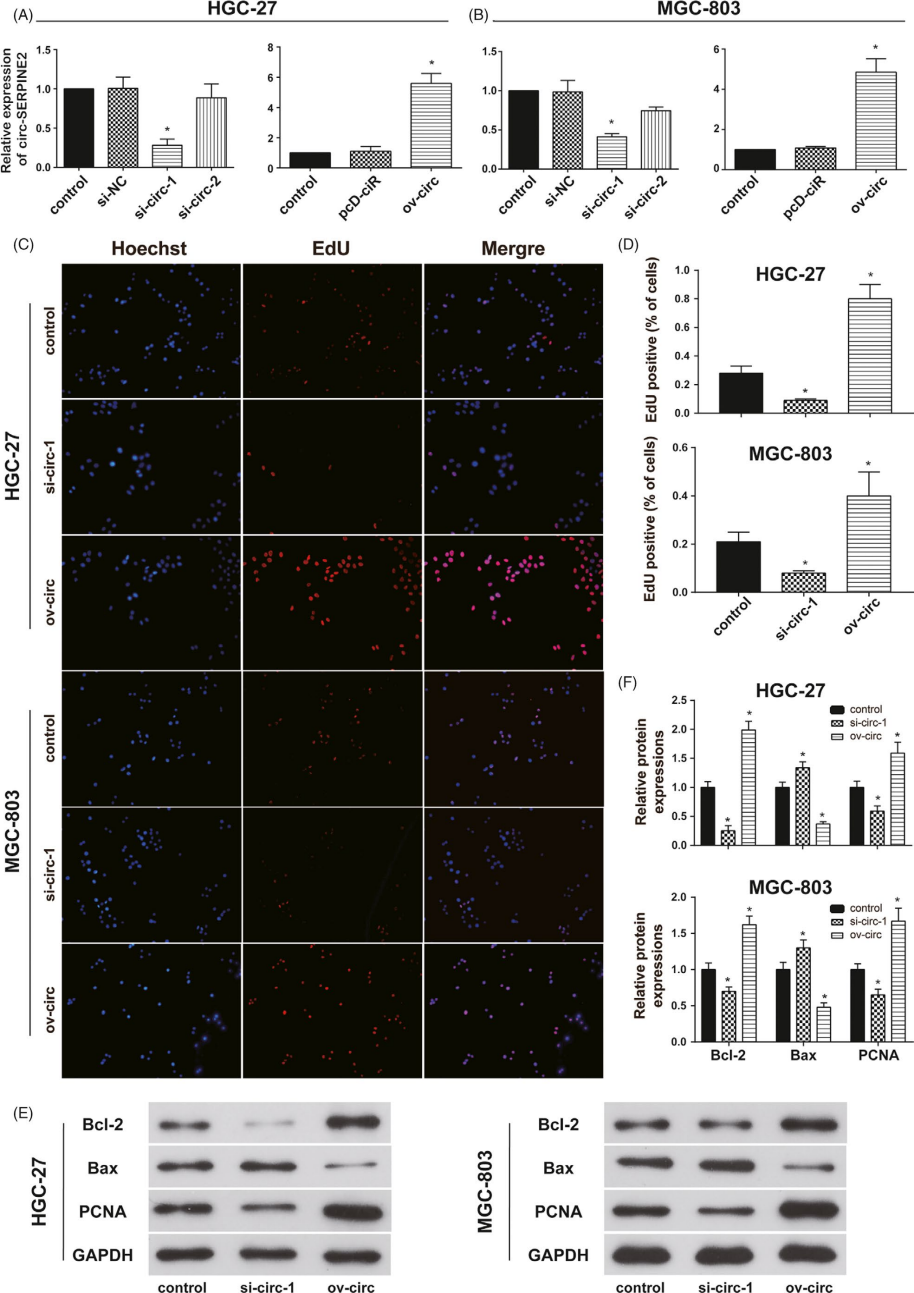
Circ‐SERPINE2 promotes cell proliferation in the GC cells. A and B, qRT‐PCR assay suggested the expression of circ‐SERPINE2 was successfully downregulated by si‐circ‐1 and upregulated by circ‐SERPINE2 overexpression vector in HGC‐27 and MGC‐803 cells. C, D, EdU assays were conducted to assess the proliferation ability of HGC‐27 and MGC‐803 cells with circ‐SERPINE2 regulation. E, F, Apoptosis and proliferation‐related proteins were detected by western blot in HGC‐27 and MGC‐803 cells with circ‐SERPINE2 regulation. Data were represented as means ± SD of at least three independent experiments. *P < 0.05

QRT‐PCR suggested circ‐SERPINE2 expressions were effectively decreased by si‐circ‐1 not si‐circ‐2 and increased by ov‐circ (Figure 3A,B). To explore the effects of circ‐SERPINE2 on proliferation and apoptosis, EdU assay (Figure 3C,D) and Western blot for proliferation and apoptosis‐related proteins' detection (Figure 3E,F) were performed. The results indicated that circ‐SERPINE2 inhibition reduced proliferation and promoted apoptosis, and circ‐SERPINE2 upregulation induced opposite results. Moreover, the correlation analysis of clinicopathological characteristics of gastric carcinoma patients and the expression of circ‐SERPINE2 indicated high expression of circ‐SERPINE2 was related to poor TNM stage (Table S3, *P* < 0.05).

**Figure 4:**
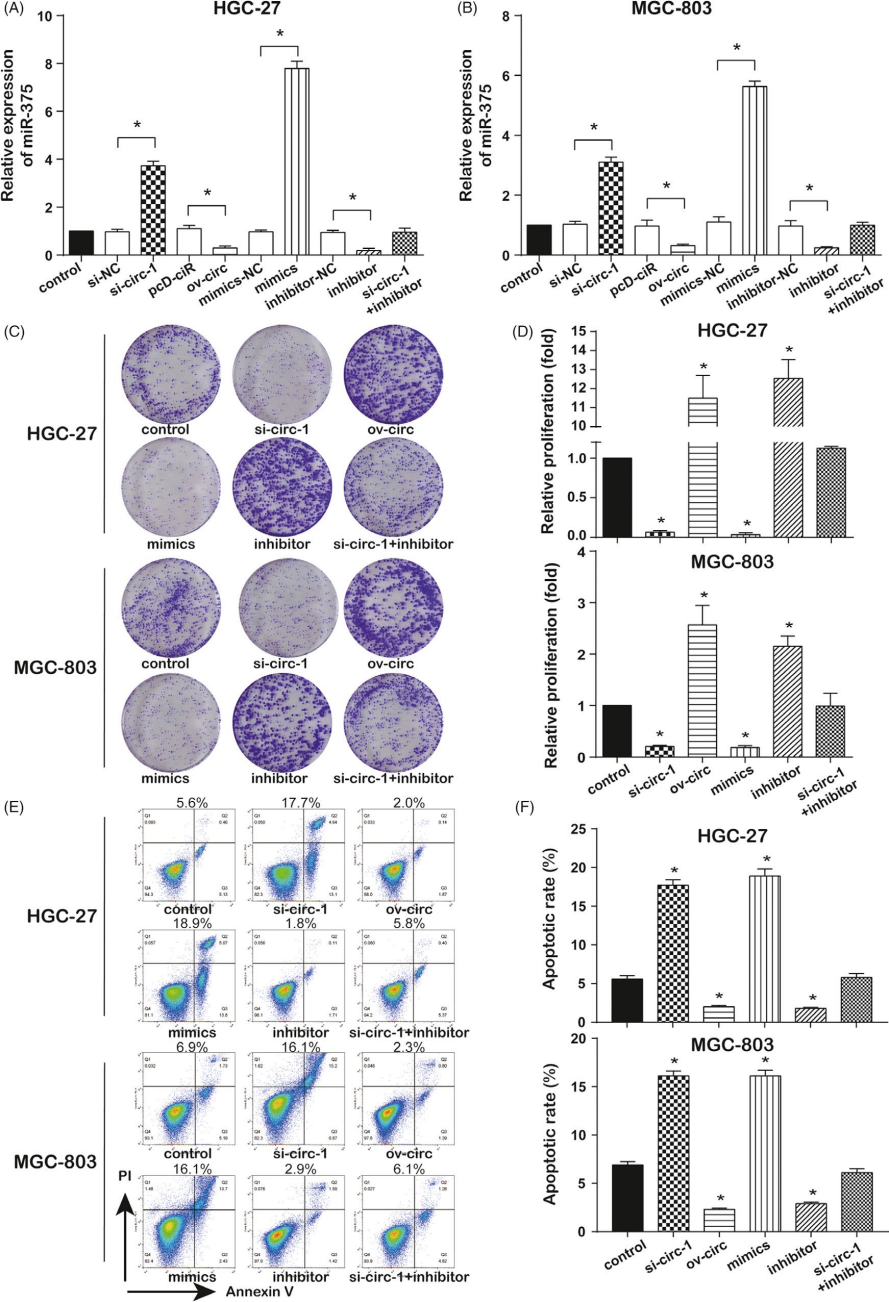
Circ‐SERPINE2 through sponging miR‐375 promotes proliferation and inhibits in the GC cells. A, B, MiR‐375 levels were measured by qRT‐PCR in HGC‐27 and MGC‐803 cells with circ‐SERPINE2 or miR‐375 regulation. C, D, Colony formation assay was performed to detect cell proliferation in HGC‐27 and MGC‐803 cells with circ‐SERPINE2 or miR‐375 regulation. E, F, Flow cytometry apoptosis analysis of HGC‐27 and MGC‐803 cells with circ‐SERPINE2 or miR‐375 regulation. Data were represented as means ± SD of at least three independent experiments. *P < 0.05

Based on the foregoing finding that circ‐SERPINE2 may exert its function through direct interaction with miR‐375, miR‐375 expression was measured after regulating circ‐SERPINE2 or miR‐375 level. The miR‐375 expression was increased by si‐circ‐1 and miR‐375 mimics and decreased by ov‐circ and miR‐375 inhibitor. However, co‐downregulating circ‐SERPINE2 and miR‐375 did not effectively change miR‐375 expression compared with control group in both HGC‐27 and MGC‐803 cells (Figure 4A,B). Colony formation assay uncovered overexpressing circ‐SERPINE2 or suppressing miR‐375 markedly enhanced cell proliferation, and downregulation circ‐SERPINE2 or upregulation miR‐375 obviously restrained cell proliferation in both HGC‐27 and MGC‐803 cells. Nevertheless, the inhibition induced by si‐circ‐1 was remitted by miR‐375 inhibitor (Figure 4C,D). Moreover, cell apoptosis was intensified by si‐circ‐1 and miR‐375 mimics and was restricted by ov‐circ and miR‐375 inhibitor in both HGC‐27 and MGC‐803 cells. Similarly, the increase in cell apoptosis caused by si‐circ‐1 was eased (Figure 4E,F). Additionally, in cell cycle assay, cell cycle progress was accelerated by ov‐circ and miR‐375 inhibitor and was held back by si‐circ‐1 and miR‐375 mimics in both HGC‐27 and MGC‐803 cells (Figure S1A,B). Taken together, our findings demonstrated that circ‐SERPINE2 promoted GC cells development by sponging miR‐375.

### 3.4 ***YWHAZ* as the downstream target gene of circ‐*SERPINE2*/miR‐375 improves GC cells**'** development**

**Figure 5:**
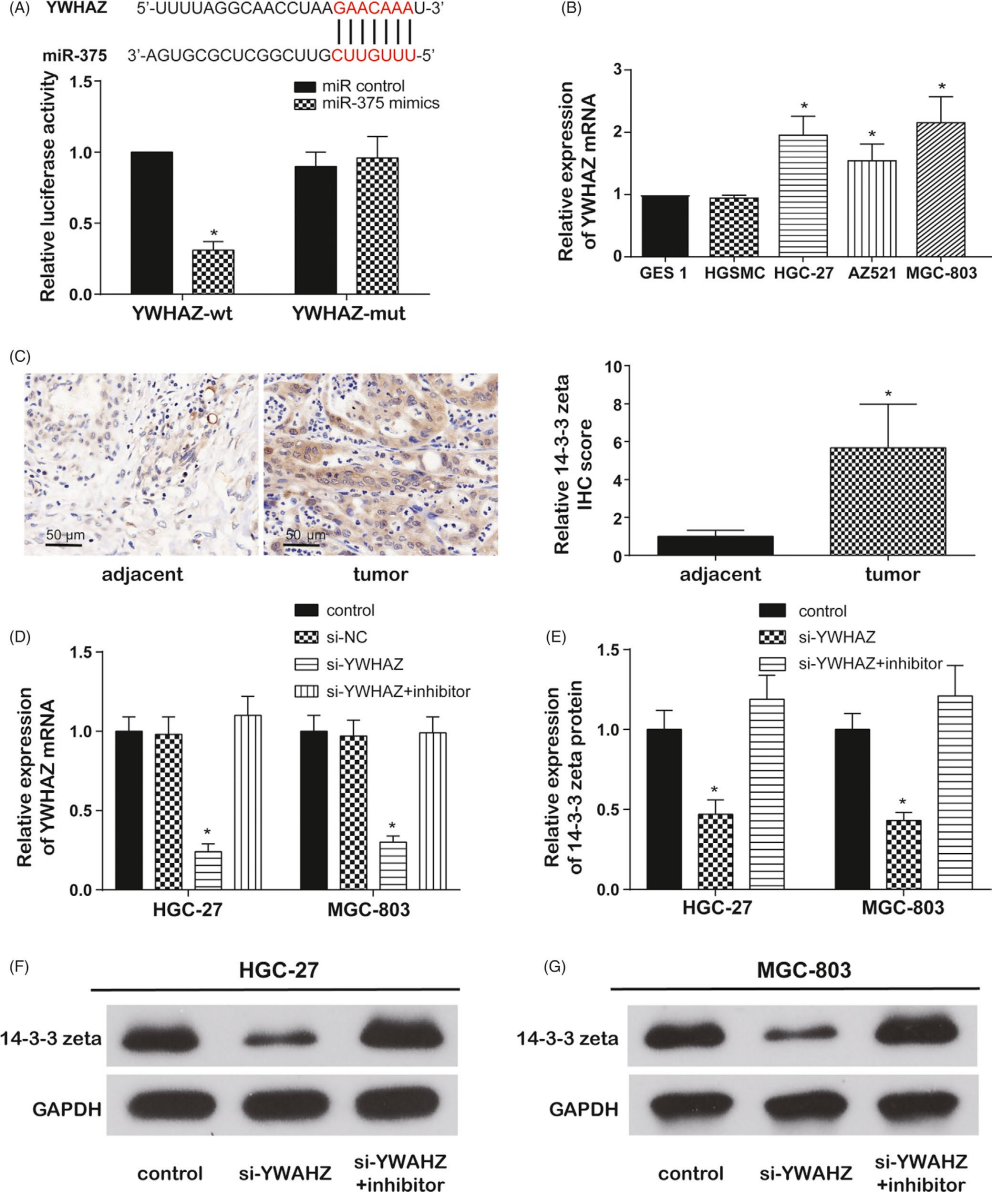
*YWHAZ* is the downstream target gene of circ‐SERPINE2/miR‐375 and upregulated in GC. A, The binding site between *YWHAZ* and miR‐375 predicted by TargetScan and dual‐luciferase reporter was performed in HEK 293T cells. B, qRT‐PCR assay suggested the expression of *YWHAZ* was upregulated in GC cell lines. C, Immunohistochemistry assay revealed 14‐3‐3 zeta protein expression was higher in GC tissue than that in the adjacent tissue. D, The expressions of *YWHAZ* were detected by qRT‐PCR after regulating *YWHAZ* or miR‐375 in HGC‐27 and MGC‐803 cells. E‐G, The protein expressions of 14‐3‐3 zeta were determined using Western blot after regulating *YWHAZ* or miR‐375 in HGC‐27 and MGC‐803 cells. Data were represented as means ± SD of at least three independent experiments. *P < 0.05

MiRecords website was utilised to indicate the validated targets of hsa‐miR‐375 and *YWHAZ*, *YAP1*, *JAK2* and *HuD* were revealed. Here, we placed emphasis on the miR‐375 target gene, *YWHAZ*, encoding 14‐3‐3 zeta protein. Firstly, TargetScanHuman 7.2 provided binding sequence of miR‐375 and *YWHAZ*, and dual‐luciferase reporter assay proved their target relationship (Figure 5A). *YWHAZ* expressions were detected using qRT‐PCR after altering circ‐SERPINE2 or miR‐375 expression in HGC‐27 and MGC‐803 cells (Figure S2). Circ‐SERPINE2 upregulation or miR‐375 inhibition markedly increased *YWHAZ* expression, circ‐SERPINE2 downregulation or miR‐375 promotion inhibited *YWHAZ* expression, and the *YWHAZ* expression was not significantly changed with co‐inhibition of circ‐SERPINE2 and miR‐375 inhibitor. Those results suggest that *YWHAZ* is the downstream target gene of circ‐SERPINE2/miR‐375.

**Figure 6:**
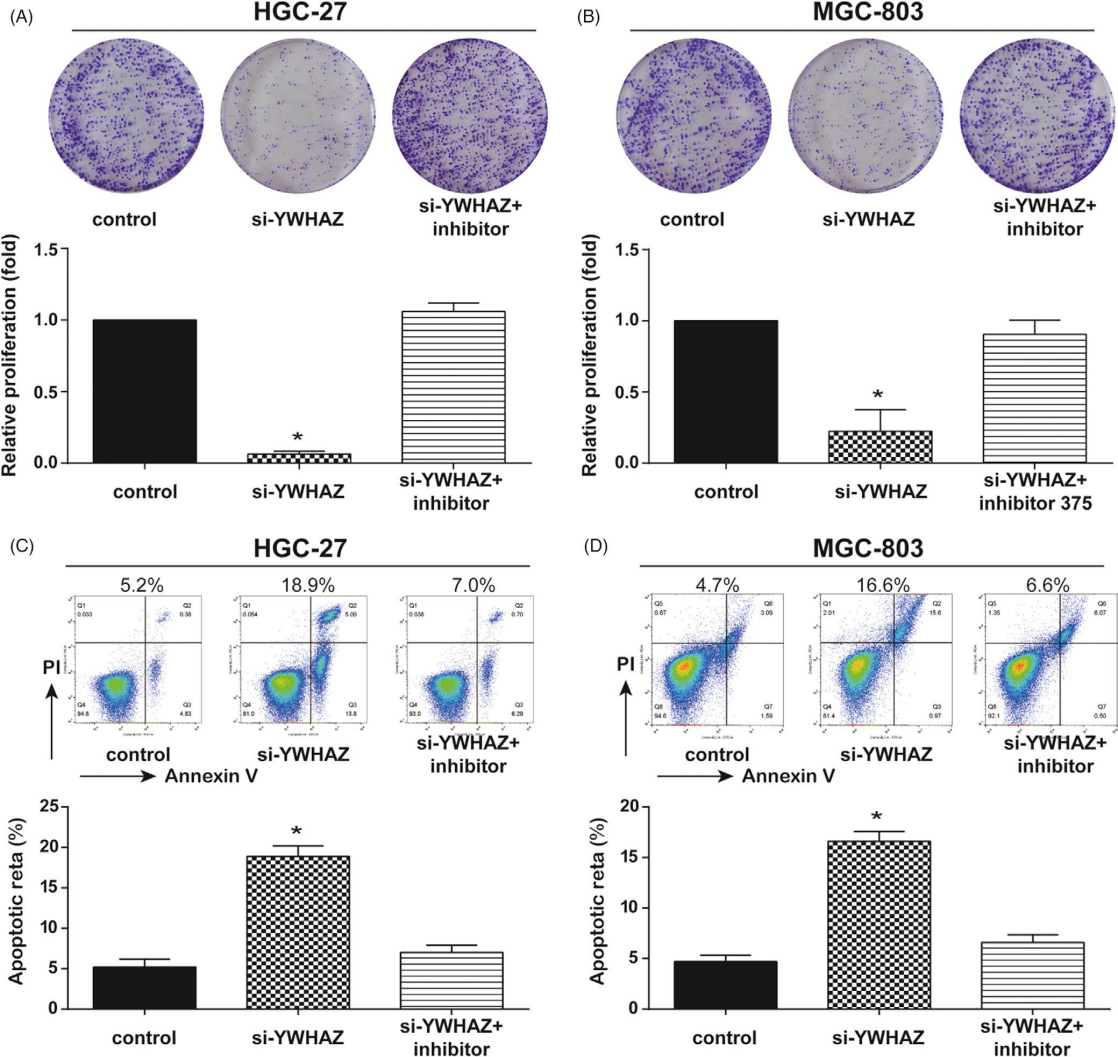
*YWHAZ* knockdown inhibits proliferation and promotes apoptosis in the GC cells. A, B, Colony formation assay was performed to detect cell proliferation in HGC‐27 and MGC‐803 cells transfected with siRNA against for *YWHAZ* or miR‐375 inhibitor. C, D, Flow cytometry was implemented to evaluate cell apoptosis in HGC‐27 and MGC‐803 cells transfected with siRNA against for *YWHAZ* or miR‐375 inhibitor. Data were represented as means ± SD of at least three independent experiments. *P < 0.05

Secondly, *YWHAZ* expressions in GC cells and control cells and its encoding protein 14‐3‐3 zeta in GC tissue and adjacent tissue were determined using qRT‐PCR and IHC. Results showed *YWHAZ* in GC cells and 14‐3‐3 zeta protein in GC tissues were significantly upregulated (Figure 5B,C). To further figure out the role of *YWHAZ* in tumour progress, HGC‐27 and MGC‐803 cells were transfected with si‐YWHAZ or co‐transfected with si‐YWHAZ and miR‐375 inhibitor. The transfection efficiency of si‐YWHAZ was confirmed in Figure 5D that si‐YWHAZ observably decreased the *YWHAZ* expression in both HGC‐27 and MGC‐803 cells. Meanwhile, miR‐375 inhibitor alleviated the decrease caused by si‐YWHAZ. The 14‐3‐3 zeta protein expression was similar to the *YWHAZ* expression after regulation *YWHAZ* or co‐regulation *YWAHZ* and miR‐375 (Figure 5E‐G). Furthermore, si‐YWHAZ repressed cell proliferation (Figure 6A,B) and cell cycle progress (Figure S3) and accelerated cell apoptosis (Figure 6C,D) in both HGC‐27 and MGC‐803 cells. Likewise, the alterations induced by si‐YWHAZ were mitigated by miR‐375 inhibitor. In conclusion, our study indicated *YWHAZ* was the downstream target gene of circ‐SERPINE2/miR‐375 and improved GC cells' development.

### 3.5 Circ‐SERPINE2 knockdown inhibits tumour growth of GC via regulation miR‐375/*YWHAZ *in vivo

**Figure 7:**
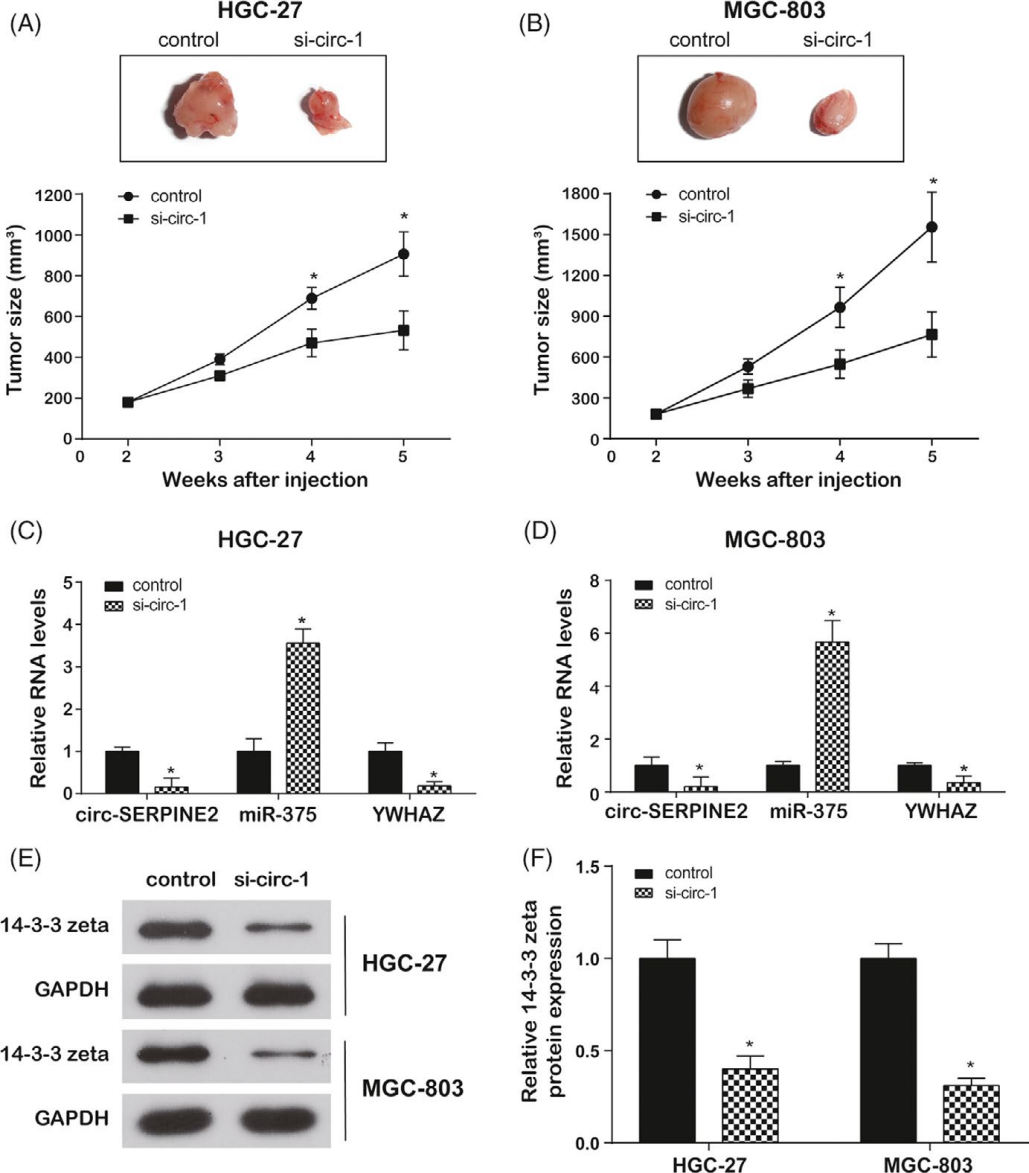
Circ‐SERPINE2 knockdown inhibits tumour growth of GC via regulation miR‐375/*YWHAZ *in vivo. A, B, The images of tumour‐bearing nude mice injected with treated HGC‐27 and MGC‐803 cells on the 35th day of injection. Tumour volumes were continually monitored with digital callipers for 4 weeks. C, D, The expressions of circ‐SERPINE2, miR‐375 and *YWHAZ* were detected using qRT‐PCR in tumours collected from nude mice. E, F, The 14‐3‐3 zeta protein levels were determined using Western blot in tumours collected from nude mice. Data were represented as means ± SD of at least three independent experiments. *P < 0.05

HGC‐27 or MGC‐803 cells (5 × 10^6^), stably transfected the mixture of si‐circ‐1 and AreloGene® or control vector, were subcutaneously injected into the right flank of nude mice. From 4 weeks after injection, the tumour volume in circ‐SERPINE2 silence group was markedly smaller than that in the control (Figure 7A,B). After continually monitoring for 4 weeks, tumours were isolated for qRT‐PCR and Western blot assays. Compared with control, circ‐SERPINE2 and *YWHAZ* expressions were lower, while miR‐375 level was higher in the si‐circ‐1 group in both HGC‐27 and MGC‐803 cells (Figure 7C,D). Similarly, 14‐3‐3 zeta protein, encoded by gene *YWHAZ,* was restrained by si‐circ‐1 (Figure 7E,F). Circ‐SERPINE2 knockdown inhibited tumour growth of GC via regulation miR‐375/*YWHAZ *in vivo.

## 4 DISCUSSION

In the current study, we found that the downregulation of circ‐SERPINE2 or the upregulation of miR‐375 could obviously restrain cell development in GC. In addition, downstream gene of circ‐SERPINE2 and miR‐375, namely *YWHAZ*, was explored in GC *YWHAZ*, targeted by miR‐375, was highly expressed in GC tissues and cells. The downregulation of *YWHAZ* inhibited cell progress in GC. Furthermore, the alterations induced by *YWHAZ* downregulation could be mitigated by miR‐375 inhibition. Therefore, in this research, we investigated the molecular mechanism of circ‐SERPINE2 in GC and explored the function of its downstream molecule for the first time.

Recent studies indicated that circRNAs might be potential biomarkers for diagnosis of GC. Hereinto, several dysregulated circRNAs were investigated in GC. Hsa_circ_0023642 was reported to be upregulated and enhanced cell migration and invasion by regulating epithelial–mesenchymal transition (EMT) in GC.^18^ The downregulated circRNAs had different effects on tumour progress in GC. Sun et al^19^ first found that hsa_circ_0000520 was observably downregulated in GC, and its level is negatively correlated with TNM stage. Li et al^20^ reported that circ_0000096 was significant low expression in GC; nevertheless, hsa_circ_0000096 knockdown significantly restrained cell migration and proliferation in vitro and in vivo. In addition, Huang et al^21^ revealed circ_0000745 was downregulated in GC and circ_0000745 level in plasma combining with carcinoembryogenic antigen (CEA) level could be regarded as a promising diagnostic marker for GC. Thus, our findings that circ‐SERPINE2 was upregulated and promoted tumour development in GC would conductive to uncover the complex function in area of circRNAs.

CircRNAs may function as competing endogenous RNAs (ceRNAs) that could sponge miRNAs to regulate miRNAs expression. CircRNA ciRS‐7 was reported to be obviously increased in GC and led to a more aggressive oncogenic phenotype by antagonising the miR‐7‐mediated PTEN‐PI3K/AKT pathway.^22^ The circHIPK3 was revealed to exert a negative regulatory effect on miR‐124/miR‐29b expression and related to Ming′s classification and T stage in GC.^23^ Besides, circular RNA_LARP4 was suggested to restrain cell proliferation and invasion in GC.^4^ Similarly, we found that the circ‐SERPINE2 expression level was high in GC and improved tumour progress by sponging miR‐375 and regulating *YWHAZ* expression.

miRNAs were believed to potentially act as valuable predictor for GC.^24^ However, the miR‐375 in the GC development exerts various roles. Smid et al^25^ analysed the expressions of 29 microRNAs in GC, of which miR‐375 was found to be high expression in GC and high level of miR‐375 was linked to a short survival. Chen et al^24^ believed that miR‐375 was decreased in GC and inhibited the advance of GC via regulating *PDK1* expression. Zhou et al^26^ proposed that miR‐375 was obviously decreased in the cisplatin‐resistant SGC7901 cells compared with the cisplatin‐sensitive SGC7901 cells. And miR‐375 upregulation significantly promoted the apoptosis‐inducing and anti‐proliferative effects of cisplatin. Zhang et al^27^ indicated that the combination of hsa‐miR‐142‐5p and hsa‐miR‐375 possessed the potential to forecast the risk of recurrence for GC patients. In our study, miR‐375 was found to target *YWHAZ*. The upregulation of miR‐375 could suppress *YWHAZ* expression to restrain gastric cancer development.

*YWHAZ* was reported to have ability to enhance the development of cancers and correlate with miR‐375 and miR‐451 in diverse cancers, including hepatocellular carcinoma (HCC),^28^ ovarian cancer,^29^ colorectal cancer (CRC),^30^ clear cell renal cell carcinoma (ccRCC)^31^ and breast cancer.^29^ Specifically, *YWHAZ* was inhibited by miR‐375 to suppress tumour properties, such as EMT, cell cycle, invasion and migration.^32^ In addition, *YWHAZ* was reported as a promoter of cisplatin and paclitaxel resistance in ovarian cancer.^28^ Similarly, our investigations demonstrated that the downregulation of *YWHAZ* could decrease the proliferation, increase the apoptosis, and extend the G1 phase of GC cells. Besides, the inhibitor of miR‐375 could counteract the influences brought by *YWHAZ*. Due to the binding relationship between miR‐375 and *YWHAZ*, it was suggested that miR‐375 directly inhibited *YWHAZ* in GC and suppressed the development of GC.

Inevitably, some limitations existed in the current research. The exploration of upstream pathways in the circ‐SERPINE2/miR‐375/*YWHAZ* axis and the correlation of circ‐SERPINE2 expression and pathological differentiation or EMT of GC need to be revealed. The clinical prognosis of patients with high or low circ‐SERPINE2 level would provide more evidences for this study. In summary, circ‐SERPINE2 was upregulated in human GC cells and tissues, and circ‐SERPINE2 promoted GC development by sponging miR‐375 and regulating *YWHAZ* expression. The axis of circ‐SERPINE2/miR‐375/YWHAZ might provide a new therapeutic target for clinical treatment of GC.

## CONFLICT OF INTEREST

All authors declare that they have no conflict of interest to declare.

## **AUTHORS**'** CONTRIBUTIONS**

JL, SL and YD substantially contributed to the conception and design of the work; DZ and WX contributed to acquisition, analysis and interpretation of the data; JL, SS and MZ drafted the manuscript; HW revising the work critically; All authors gave final approval of the work.

## ETHICAL APPROVAL

All procedures performed in studies involving human participants were in accordance with the ethical standards of The Second Hospital of Shandong University committee. Informed consent was obtained from all individual participants included in the study. All procedures involving animals were performed in compliance with guidelines of The Second Hospital of Shandong University.

## DATA AVAILABILITY STATEMENT

The data that support the findings of this study are available from the corresponding author upon reasonable request.
