# Supplementary figures and images for: Circ‐SERPINE2 promotes the development of gastric carcinoma by sponging miR‐375 and modulating YWHAZ
Source: Cell Prolif. 2019 Jun 14;52(4):e12648. doi: 10.1111/cpr.12648 (PMC6668981; doi:10.1111/cpr.12648)

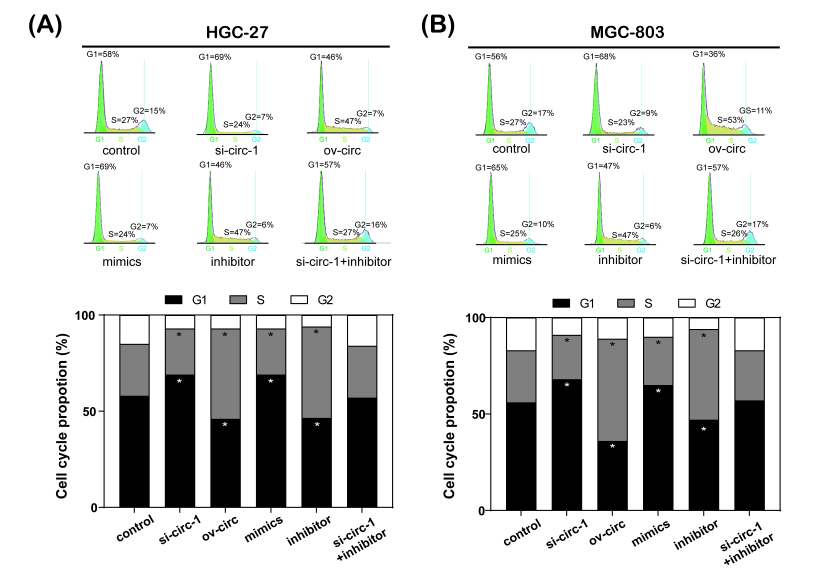

Supplement: Supplementary file 1 [file CPR-52-e12648-s001.tif]

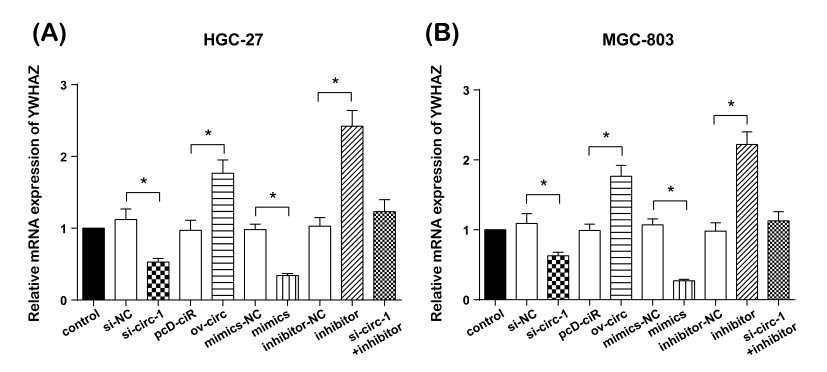

Supplement: Supplementary file 2 [file CPR-52-e12648-s002.tif]

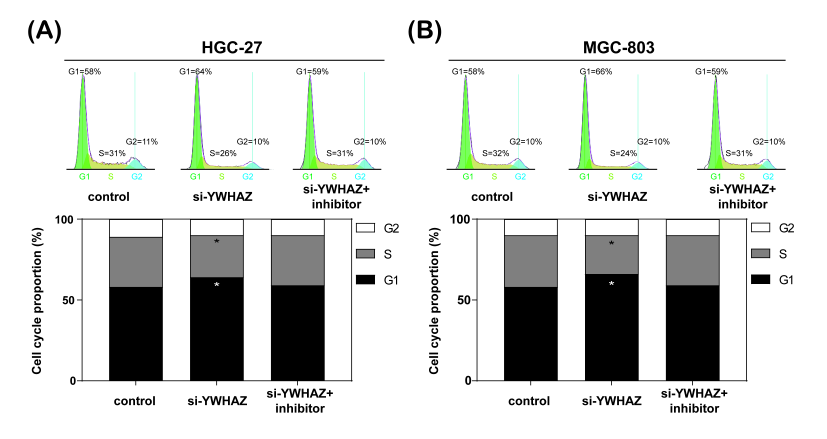

Supplement: Supplementary file 3 [file CPR-52-e12648-s003.tif]
