# Supplementary material for: Circ‐SERPINE2 promotes the development of gastric carcinoma by sponging miR‐375 and modulating YWHAZ
Source: Cell Prolif. 2019 Jun 14;52(4):e12648. doi: 10.1111/cpr.12648 (PMC6668981; doi:10.1111/cpr.12648)
Supplement: Supplementary file 4 [file CPR-52-e12648-s004.docx]

**Supplementary Table S1 Differentially expressed circRNAs both in GSE78092 and GSE93541**

|  | **GSE78092** |  |  |  |  | **GSE93541** |  |  |
| --- | --- | --- | --- | --- | --- | --- | --- | --- |
| genesymbol | logFC | *P*.Value | adj.*P*.Val | | genesymbol | logFC | *P*.Value | adj.*P*.Val |
| hsa_circ_0000507 | -1.19861 | 0.000415 | 0.020096 |  | hsa_circ_0000507 | -1.211232 | 0.009498 | 0.046325 |
| hsa_circ_0008365 | 1.49433 | 0.000902 | 0.028162 |  | hsa_circ_0008365 | 1.659112 | 0.000709 | 0.009633 |
| hsa_circ_0067127 | 1.45094 | 7.70E-05 | 0.008619 |  | hsa_circ_0067127 | 1.825122 | 0.000745 | 0.009814 |

logFC: log(FoldChange_Tumor/Normal); “-” means down-regultation in tumor; “adj.*P*.Val < 0.05” means a statistical significance.
