# Supplementary material for: Circ‐SERPINE2 promotes the development of gastric carcinoma by sponging miR‐375 and modulating YWHAZ
Source: Cell Prolif. 2019 Jun 14;52(4):e12648. doi: 10.1111/cpr.12648 (PMC6668981; doi:10.1111/cpr.12648)
Supplement: Supplementary file 5 [file CPR-52-e12648-s005.docx]

**Supplementary Table S2 CircRNAs**' **information got from web of CircInteractome**

| **CircRNA ID** | **hsa_circ_0067127** | | **hsa_circ_0000507** | | **hsa_circ_0008365** | |
| --- | --- | --- | --- | --- | --- | --- |
| Genomic Length | 13597 bp | | 235 bp | | 10120 bp | |
| Spliced Seq Length | 812 bp | | 235 bp | | 707 bp | |
| Best Transcript | NM_012190 | | NM_001008895 | | NM_006216 | |
| Location | chr3:125843206-125856803 | | chr13:113869721-113869956 | | chr2:224856519-224866639 | |
| Gene Symbol | ALDH1L1 | | CUL4A | | SERPINE2 | |
|  |  | |  | |  | |
| RNA-binding protein sites matching flanking regions of circRNA | RNA-binding Protein | # Tags | RNA-binding Protein | # Tags | RNA-binding Protein | # Tags |
|  | FUS | 1 | EIF4A3 | 6 | AGO2 | 1 |
|  |  |  | FMRP | 1 | EIF4A3 | 5 |
|  |  |  | FUS | 1 |  |  |
|  |  |  | HNRNPC | 1 |  |  |
|  |  |  | TDP43 | 6 |  |  |
