# Supplementary material for: Circ‐SERPINE2 promotes the development of gastric carcinoma by sponging miR‐375 and modulating YWHAZ
Source: Cell Prolif. 2019 Jun 14;52(4):e12648. doi: 10.1111/cpr.12648 (PMC6668981; doi:10.1111/cpr.12648)
Supplement: Supplementary file 6 [file CPR-52-e12648-s006.docx]

**Supplementary Table 3 clinicopathological characteristics of 49 gastric carcinoma patients and the expression of circ-SERPINE2**

| Parameters | Group | cases | Circ-SERPINE2 | | *P*-value |
| --- | --- | --- | --- | --- | --- |
|  |  |  | High, n (%) | Low, n (%) |  |
| Age at surgery (year) | <55 | 31 | 18 (58.06%) | 13 (41.94%) | >0.9999 |
|  | ≥55 | 17 | 10 (55.56%) | 7 (44.44%) |  |
| Sex | Female | 19 | 12 (63.16%) | 7 (36.84%) | 0.5636 |
|  | Male | 30 | 16 (53.33%) | 14 (46.67%) |  |
| Diameter in cm | ≥5 | 17 | 12 (70.59%) | 5 (29.41%) | 0.2291 |
|  | <5 | 32 | 16 (50.00%) | 16 (50.00%) |  |
| Lymphatic metastasis | Present | 35 | 23 (65.71%) | 12 (34.29%) | 0.1083 |
|  | Absent | 14 | 5 (35.71%) | 9 (64.29%) |  |
| TNM stage | I-II | 15 | 4 (26.67%) | 11 (73.33%) | 0.0058* |
|  | III-IV | 34 | 24 (70.59%) | 10 (29.41%) |  |

**P* < 0.05 represents statistical significance (Chi-square test or Fisher′s exact test).
